# Community violence exposure and substance use: cross-cultural and gender perspectives

**DOI:** 10.1007/s00787-017-1097-5

**Published:** 2017-12-20

**Authors:** Sandra Löfving-Gupta, Mimmie Willebrand, Roman Koposov, Marek Blatný, Michal Hrdlička, Mary Schwab-Stone, Vladislav Ruchkin

**Affiliations:** 1 Child and Adolescent Psychiatry Unit, Department of Neuroscience, Uppsala University, 751 85 Uppsala, Sweden; 2 Regional Centre for Child and Youth Mental Health and Child Welfare, UiT The Arctic University of Norway, Tromsö, Norway; 3 Institute of Psychology, Academy of Sciences of the Czech Republic, Brno, Czech Republic; 4 Department of Child Psychiatry, Charles University Second Faculty of Medicine, University Hospital Motol, Prague, Czech Republic; 5 Child Study Center, Yale University Medical School, New Haven, CT 06520 USA; 6 Säter Forensic Psychiatric Clinic, 783 27 Säter, Sweden

**Keywords:** Community violence exposure, Substance use, Gender, Adolescents

## Abstract

The negative effects of community violence exposure on child and adolescent mental health are well documented and exposure to community violence has been linked both to a number of internalizing and externalizing symptoms. Our aim was, therefore, to investigate cross-cultural and gender differences in the relationship between community violence exposure and substance abuse. A self-report survey was conducted among 10,575, 12–18 year old adolescents in three different countries, Czech Republic (*N* = 4537), Russia (*N* = 2377) and US (*N* = 3661). We found that in all three countries both substance use and problem behavior associated with it increased similarly along with severity of violence exposure and this association was not gender-specific. It was concluded that in spite of the differences in the levels of violence exposure and substance use cross-culturally and by gender, the pattern of their association is neither culturally nor gender bound.

## Introduction

Although the rates of community violence exposure have declined since the peak in the 90s, it is still regarded as a “major public health problem” [1]. WHO has declared violence” a leading worldwide health problem” [2], and yet, most research regarding community violence exposure has focused on high-risk youth in the United States [3–5] giving a rather narrow and perhaps, even skewed picture of the consequences of violence exposure.

The negative effects of community violence exposure on child and adolescent mental health are well documented and exposure to community violence has been linked both to a number of internalizing symptoms, such as anxiety, depression and somatization, as well as externalizing symptoms, like aggression and delinquency (for a review see e.g. [4, 6, 7]). Some studies have also explored the role of substance abuse, as a possible outcome of violence exposure [8–11] linking the exposure to both magnitude and frequency of substance abuse. However the effect of violence exposure on substance use is far less examined [12].

It has been suggested that the effects of community violence can be long-lasting and can impact psycho-social functioning of youth in years after the exposure took place, which has been demonstrated by a number of longitudinal studies [6, 13]. Several factors have been identified that can potentially influence the extent and the consequences of traumatic exposure, including some aspects of trauma itself, age, and gender of the victim. The degree of exposure and proximity to trauma both influence psychopathology, developing after traumatic exposure, with a stronger association found in relation to victimization, as compared to witnessing [4]. When examining the consequences of violence exposure, it is also important to consider the fact that some youth may report higher levels of violence exposure by virtue of themselves being involved in violence as perpetrators and traumatic response among perpetrators might differ from that of innocent bystanders [13]. Antisocial youth may differ from others both in terms of their reaction to trauma, as well as in terms of higher levels of other externalizing problems, such as substance abuse.

There is still a certain controversy regarding gender specific effects of traumatic experience. While some studies suggest that girls are less exposed to community violence, as compared to boys [14], others found that females are more likely to experience trauma than males [15]. It has also been suggested that boys tend to react with more externalizing behaviors, whereas the girls to a higher degree respond with internalizing symptoms following exposure to community violence [13]. Age is another important factor both concerning the prevalence of exposure as well as the effects of it, where older children tend to report a higher prevalence [5] and seem to react with more externalizing symptoms compared to younger ones [4].

However, questions remain about the generalizability of the above findings to different cultures. There is some evidence that socio-cultural factors can influence both the prevalence and nature of symptoms. Some argue that the adverse effects of trauma exposure may differ by culture/ethnicity and that the expression of emotional distress is culture bound [16, 17], whereas others found that response to trauma is universal [18]. There are also differences in how symptoms are described and the explanatory models tend to vary across cultures, with attribution of symptoms to medical illness, family, work, environmental stress or culture-specific phenomena [19].

In summary, the mental health outcomes of community violence exposure are by now well established, however it’s relationship to substance use as well as how this relationship varies by gender and culture is poorly examined. The present study builds on one of the few studies on community violence with a cross-cultural perspective [10] however using a larger, culturally different sample and adding a gender perspective. The present study hypothesized that increased severity of exposure (from no exposure to witnessing to victimization) is associated with an increase in substance abuse, even when controlling for own perpetration of violence and for emotional problems. We further hypothesized that these relationships may be gender-specific, with higher levels of substance use in relation to community violence exposure among boys.

## Method

### Participants and procedure

Data in this study were drawn from the Social and Health Assessment (SAHA) conducted in the Czech Republic, Russia, and the US in the spring of 2003. The primary aim of this study was to determine the factors associated with adolescent health and well-being. The study sites were the following: Russia [the city of Arkhangelsk (population 360,000)]; the US [the city of New Haven, Connecticut (population 125,000)]; and the Czech Republic [the capital Prague (population 1.2 million) and all 12 regional capitals (population 50,000–400,000)]. Details of the survey and its methodology have been previously published elsewhere [20, 21]. In brief, in New Haven, all students aged 12–17 who were in the public school system were included In Arkhangelsk and the Czech study locations, data were collected from a representative sample of students aged 12–17 and 12–18, respectively, in all public schools. Students were recruited from within classes that were randomly selected from within schools that had themselves been randomly selected (excepting New Haven, where all students were included). In all countries, students completed the survey in their classrooms during a normal school day. The survey was administered by research project staff and by school system personnel in sessions lasting 45–60 min. All administrators were trained in a standardized procedure for administering the survey. Each student received a survey booklet, which contained all of the questions and response options. Students followed along the survey, reading questions to themselves and circling responses in the booklet. Written informed consent was obtained from all participants prior to the survey administration, and both parents (for their children) and children had the right to refuse to participate. All students were surveyed unless their parents requested that they not be involved, the students said that their parents had objections, or the students said that they themselves wished not to participate. Response rates for these surveys were high with only 3.6% of children refusing to participate in Russia, 1.4% in the Czech Republic and < 1% in the United States. For comparability, the present study is limited to those adolescents who were aged 12–18 years old with the analytical sample thus comprising 4537 adolescents from the Czech Republic, 2377 from Russia and 3661 from the United States.

### Ethical approval

Ethical approval for the study was obtained from the Northern State Medical University in Arkhangelsk, Yale University School of Medicine and the Institute of Psychology, Academy of Sciences of the Czech Republic, v.v.i.

### Measures

#### The Social and Health Assessment

The Social and Health Assessment (SAHA), a survey (self-report questionnaire) developed by Weissberg et al. [22] and adapted by Schwab-Stone et al. [6, 23], served as the basis for this study. All instruments, described below, were used as parts of the survey, and included both new scales developed specifically for the survey and scales available from the literature that has been used with similar populations.

*Witnessing and victimization* Items assessing witnessing and victimization were derived from the Screening Survey of Exposure to Community Violence, developed by Richters and Martinez [24]. The students were asked “about things that may happen to people in some neighborhoods”. Using 5-point scale response format (ranging from “None” (0) to “10 or more times” (4)) the students described whether they had in the past year witnessed or been victimized by six types of violence (been beaten up or mugged, threatened with serious physical harm, shot or shot at with a gun, attacked or stabbed with a knife, chased by gangs or individuals, or seriously wounded in an incident of violence). Three groups were formed according to the reported types of exposure. Those who did not report any witnessing and victimization episodes were considered as the *non*-*exposed group*. Those, who reported at least one episode of witnessing, but no episodes of victimization were considered as the *witnessing group*. Finally, those, who reported at least one episode of victimization were considered the *victimization group*.

*Substance use* Items on alcohol use were derived from the Monitoring the Future Scale [25] and for cigarette use, marijuana use, and hard drug use from the School Health Study [26]. Cigarette use, henceforth referred to as smoking, was assessed by 3 items that asked whether the respondent had ever smoked cigarettes, how frequently the respondent had smoked during the last 30 days, and how many cigarettes he or she smoked daily during the last 30 days. Each item had a 4-point response scale, and all 3 were summed to obtain a total smoking score. Alcohol use was assessed by a total of 4 items that addressed consumption during the past 30 days. Three items addressed the use of 3 different alcoholic beverages [How many times (if any) have you had a drink of… (beer, wine, hard liquor)] on a 4-point scale (0, 1–2, 3–5, 6 or more times), and 1 item assessed the frequency of binge drinking [On how many days (if any) did you have five or more drinks of alcohol in a row, that is, within a couple of hours?] on a 5-point scale (0, 1, 2, 3–5, 6 or more times). A “drink” was defined as a bottle or can of beer, a glass of wine or wine cooler, a shot of liquor, or a mixed drink. Three items addressing the use of alcoholic beverages were summed to obtain a total alcohol consumption index in the past 30 days. The binge drinking in the past 30 days was assessed separately. Marijuana use was assessed by 2 questions on lifetime use and use during the past 30 days, using a 4-point scale. The items scores were summed in order to obtain a marijuana use index. Problems related to substance use (lifetime) were assessed by five items [27] asking whether the respondent ever had problems related to the use of drugs, such as getting into an argument, feeling sick, getting arrested, or having money problems. The items are rated on a three-point scale (from “Never” to “Often”).

*Violent and aggressive behavior* of the respondents in the past 2 years was assessed by six items from the SAHA, including starting a fist fight or shoving match, hurting someone so badly in a fight that treatment by a doctor was needed, carrying a gun, being in a gang or posse fight, being arrested by the police, and carrying a blade, knife or gun in school. These six items were selected from a larger pool of conduct problems and antisocial behavior items to reflect the types of violence also assessed by the witnessing and victimization measures. The selection of these 6 items was further supported by factor analysis. Responses were tallied on a 4-point scale, producing a total score range from 0 to 24. The scale had an adequate internal consistency (Cronbach *α* = 0.81).

*Emotional symptoms* were assessed using the corresponding scale from the Strength and Difficulties Questionnaire (SDQ), which is a brief behavioral screening questionnaire about 4–16 year olds [28], which has demonstrated good validity in identifying psychiatric problems in children [29]. The emotional symptoms scale consists of five items, assessing child’s negative feelings for the past 6 months on a three-point scale [Not True (0), Somewhat True (1), Certainly True (2)] (Cronbach *α* = 0.70).

*Proxy for Socioeconomic Status (SES)* was based on the student reports of coming from a single-parent family (1 vs. 0) and of parental employment status (calculated for each parent separately), (2 = unemployed, 1 = part-time employed, 0 = full-time employed). A proxy for SES was calculated as a sum of the above variables (single-parent family status and parental employment status), hence producing a variable potentially ranging from 0 to 5.

### Statistical analyses

Data were analyzed using the Statistical Package for Social Sciences (SPSS-22.0). Although direct between-country comparisons of the prevalence of violence exposure may seem unjustified, due to differences in culture, ethnicity, socioeconomic status, size of the city and other parameters, we nevertheless provide the basic rates of exposure to violence (both by the type of exposure and a summary table) for each study group, as we hypothesized that in spite of expected differences in the levels of exposure and substance use, there would be specific patterns of relationships, potentially generalizable across the three contexts.

Multivariate analyses of covariance (MANCOVA) were performed in order to assess differences in the levels of alcohol and substance use in boys and girls, who had experienced violence exposure of different severity (no violence exposure, witnessing and victimization). Hence, we used 3 (violence exposure) × 3 (country) × 2 (gender) design for the substance use scores. Because demographic characteristics, such as age and SES, as well as the own involvement in violent behavior and own emotional problems influence children’s developmental process and outcomes variables, all analyses were conducted controlling for age, SES, emotional problems and for own violent behavior score.

## Results

Table 1 shows the prevalence of violence exposure by country and gender. Similar to the previous studies, students from all three countries reported relatively high prevalence of exposure to community violence. Within each study group, more boys were exposed to episodes of violence than girls. The proportion of boys and girls in Russia and Czech Republic who witnessed at least one episode of violence was similar within country, but in the US a higher proportion of girls than boys reported witnessing violent events. In all three countries, more boys reported episodes of victimization by violence than girls (Table 1).

**Table 1:** Within-country comparisons of community violence exposure rates by gender [*N* (%)]

	Czech Republic		Russia		US	
	Boys	Girls	Boys	Girls	Boys	Girls
No exposure	599 (29.0)	1051 (39.3)	356 (29.9)	671 (41.1)	394 (19.6)	513 (24.5)
Witnessing	708 (34.3)	1079 (40.3)	447 (37.6)	670 (41.0)	777 (38.7)	1087 (51.9)
Victimization	759 (36.7)	547 (20.4)	387 (32.5)	292 (17.9)	837 (41.7)	493 (23.6)
Statistics	*χ* ^2^ = 159.19, *p* < 0.001		*χ* ^2^ = 87.05, *p* < 0.001		*χ* ^2^ = 154.45, *p* < 0.001	

Table 2 presents the descriptive statistics (M, SD) for MANCOVA regarding differences in substance use according to the degree of severity of violence exposure for boys and girls in three countries. Table 3 presents effect sizes for each dependent variable (alcohol use and substance use scales), as well as the summary statistics. The main effect for the degree of exposure for the total group was significant (Wilks’ lambda = 0.966; *F*(10, 21,098) = 36.33, *p* < 0.000, *η* ^2^ = 0.017), with increasing substance use scores for increasing exposure to community violence. The main effect for Gender was significant (Wilks’ lambda = 0.994; *F*(5, 10,549) = 12.71, *p* < 0.000, *η* ^2^ = 0.006), demonstrating a difference in substance use between boys and girls. The main effect for Country was significant (Wilks’ lambda = 0.788; *F*(10, 21,098) = 266.48, *p* < 0.000, *η* ^2^ = 0.112), suggesting differences in baseline levels of substance use in these three samples. Also, the interaction effect for Country × gender was significant (Wilks’ lambda = 0.988; *F*(10, 21,098) = 12.38, *p* < 0.000, *η* ^2^ = 0.006), suggesting that gender differences in substance use followed different patterns in different cultures. The interaction effect for degree of exposure × Country was also significant (Wilks’ lambda = 0.972; *F*(20, 34,988) = 15.13, *p* < 0.000, *η* ^2^ = 0.007), suggesting that the substance use levels differ depending on the level of violence exposure and country. However, neither the interaction effect for degree of exposure × gender (Wilks’ lambda = 0.999; *F*(10, 21,098) = 1.49, ns, *η* ^2^ = 0.001), nor the interaction effects for degree of exposure × Country × gender (Wilks’ lambda = 0.997; *F*(20, 34,988) = 1.46, ns, *η* ^2^ = 0.001) were significant, suggesting that patterns of substance use in response to varying degree of violence exposure were not gender-specific, and that despite substantial differences in the levels of substance use by country, by gender, and by varying degree of exposure to violence the gender-specific patterns of response to community violence exposure were similar across the three samples. In addition, the main effects for age (Wilks’ lambda = 0.858; *F*(5, 10,549) = 349.49, *p* < 0.000, *η* ^2^ = 0.142), for own violent behavior (Wilks’ lambda = 0.853; *F*(5, 10,549) = 363.11, *p* < 0.000, *η* ^2^ = 0.147), for emotional problems (Wilks’ lambda = 0.997; *F*(5, 10,549) = 5.93, *p* < 0.000, *η* ^2^ = 0.003), and for SES proxy (Wilks’ lambda = 0.998; *F*(5, 10,549) = 4.80, *p* < 0.000, *η* ^2^ = 0.002), were all significant and suggesting increasing use of substances with age, with increased involvement in violent behavior and with increased levels of emotional problems, as well as with lower SES. As shown in Table 3, in the three samples, substance use scores increased with severity of violence exposure and, as demonstrated by the follow-up univariate effects for degree of exposure, those who were victimized by violence reported the highest levels of substance use.

**Table 2:** Descriptive statistics regarding substance use scores [*M* (SD)] in Czech Republic, Russia and the US by degree of exposure in boys (B) and girls (G)

	Non-exposed	Witnessing	Victimization
Alcohol use			
Czech			
B	1.48 (2.17)	2.56 (2.70)	3.07 (2.91)
G	1.33 (1.97)	2.37 (2.46)	2.63 (2.63)
Russia			
B	1.54 (2.22)	2.69 (2.79)	3.28 (2.87)
G	1.59 (1.91)	2.55 (2.47)	3.14 (2.68)
US			
B	0.37 (1.27)	0.58 (1.48)	1.39 (2.30)
G	0.44 (1.08)	0.82 (1.67)	1.39 (2.14)
Binge drinking			
Czech			
B	0.43 (0.99)	0.94 (1.39)	1.13 (1.43)
G	0.36 (0.85)	0.71 (1.18)	0.95 (1.27)
Russia			
B	0.43 (0.90)	0.91 (1.30)	1.19 (1.37)
G	0.47 (0.85)	0.95 (1.26)	1.36 (1.43)
US			
B	0.07 (0.45)	0.13 (0.56)	0.42 (0.96)
G	0.09 (0.41)	0.18 (0.64)	0.40 (0.91)
Cigarette use			
Czech			
B	1.66 (2.33)	2.72 (2.97)	3.63 (3.23)
G	2.01 (2.58)	3.32 (3.09)	4.12 (3.29)
Russia			
B	2.23 (3.04)	3.80 (3.43)	4.59 (3.46)
G	2.17 (2.72)	3.23 (3.20)	4.29 (3.38)
US			
B	2.36 (1.06)	2.62 (1.47)	3.36 (2.24)
G	2.71 (1.48)	3.08 (1.89)	3.80 (2.37)
Marijuana use			
Czech			
B	0.51 (1.22)	1.17 (1.87)	1.68 (2.15)
G	0.54 (1.27)	1.14 (1.74)	1.53 (2.01)
Russia			
B	0.14 (0.64)	0.33 (1.04)	0.43 (1.15)
G	0.10 (0.41)	0.16 (0.56)	0.29 (0.91)
US			
B	1.18 (0.79)	1.47 (1.30)	2.04 (1.85)
G	1.27 (0.94)	1.50 (1.27)	1.96 (1.77)
Substance-use-related problems			
Czech			
B	0.17 (0.56)	0.47 (0.93)	0.71 (1.25)
G	0.19 (0.57)	0.46 (0.91)	0.73 (1.21)
Russia			
B	0.08 (0.73)	0.18 (0.72)	0.40 (1.16)
G	0.07 (0.52)	0.07 (0.49)	0.26 (1-04)
US			
B	0.09 (0.51)	0.21 (0.74)	0.79 (1.74)
G	0.12 (0.55)	0.24 (0.73)	0.62 (1.31)

The values presented are not adjusted for the list of covariates

**Table 3:** Effect sizes for each dependent variable and summary statistics (*η* ^2^, *p*)

	Alcohol use	Binge drinking	Cigarette use	Marijuana use	Substance-use-related problems
Violence exposure	0.020, < 0.001	0.017, < 0.001	0.027, < 0.001	0.010, < 0.001	0.006, < 0.001
Country	0.040, < 0.001	0.030, < 0.001	0.010, < 0.001	0.077, < 0.001	0.008, < 0.001
Gender	0.002, < 0.001	0.002, < 0.001	0.005, < 0.001	0.001, < 0.001	0.003, < 0.001
Violence exposure by country	0.005, < 0.001	0.008, < 0.001	0.008, < 0.001	0.009, < 0.001	0.004, < 0.001
Violence exposure by gender	0.000, ns	0.001, < 0.05	0.001, ns	0.000, ns	0.000, ns
Country by gender	001, < 0.05	0.003, < 0.001	0.003, < 0.001	0.000, ns	0.001, < 0.05
Violence exposure by country by gender	0.000, ns	0.001, ns	0.000, ns	0.000, ns	0.001, ns

Considering that the differences by outcome, country and gender could have been masked by use of the MANCOVA analysis (i.e. by simultaneously assessing all three outcomes in one model), we have also attempted to examine each outcome separately in order to determine whether the patterns that are reported from the MANCOVA hold up with each outcome individually. The results obtained have been largely similar.

## Discussion

This cross-sectional, cross-cultural study found a significant relation between exposure to community violence on the one side, and alcohol and substance use on the other side, and this association seems to be robust across culture and gender. Across all three countries boys were more often victimized by violence than girls. After controlling for the subjects own level of violent behavior and for emotional problems, levels of alcohol and substance use increased along with increased level of violence exposure. Although, this association seems in some ways to be gender-specific (i.e. as concerns binge drinking), the pattern of these associations seems to hold in all three cultures.

The relation between violence and emotional problems on the one side and violence exposure and substance use on the other side is intricate and seems to be suggestive rather than conclusive, as they all represent risk factors for one another and may often co-occur [30, 31]. Some theories suggest that engagement in high-risk behaviors, like substance use, may represent a way of coping with negative effects following violence exposure for adolescents [32]. From this perspective, substance use has been considered as a “self-medication” in order to cope with the aftermath of violence exposure [9, 31]. This hypothesis is further supported by a prospective study of young adults that concluded that individuals with PTSD had four times higher risk of developing substance use disorder than those without the diagnosis [33]. At the same time, youth who use drugs tend to spend more time in risky environments and hence, are at greater risk for victimization by community violence [28]. Furthermore, substance use is connected to violent behavior either directly though disinhibition, or indirectly through association with aggressive peers, reduced coping skills, and increased life stressors [34]. In addition, those youth who perpetrate violence themselves are also more likely to witness or become victims of violence [35]. Hence, controlling for own violent behavior and emotional problems is essential when examining the relationship between violence exposure and substance abuse.

The present study found that in all three countries substance use increases along with the degree of violence exposure from no exposure, to witnessing to victimization. Previous studies have found a strong association between witnessing community violence and substance use [9] and one longitudinal study even found that this association was stronger with witnessing compared to victimization [36]. Assuming that the patterns of psychopathology in perpetrators and innocent bystanders tend to differ, one explanation for this discrepancy might be that the later study did not control for the subjects own violent acts.

Although, the research on gender differences with regard to the prevalence and consequences of community violence exposure has been rather inconclusive, several American studies have reported higher levels of exposure to community violence among boys than girls [5, 37] with more pronounced differences for victimization rates, as compared to witnessing. Our findings of boys reporting more episodes of victimization, as compared to girls, support the previous research and seem to hold across different countries, except for the high number of girls in the US sample reporting witnessing violence, as compared to boys.

There have been only a handful of studies regarding gender differences in the association between violence exposure and substance use and the findings tend to be inconclusive. One longitudinal study, Pinchevsky et al. [36], found that substance use in girls (but not in boys) was more strongly associated with witnessing, as opposed to direct victimization and that effect of victimization on binge drinking was also stronger in girls than in boys. However, Kilpatrick et al. [31], found no significant gender differences in the effect of witnessing violence on alcohol and drug use in youth, and neither did Kaufman [38], when examining the associations between victimization by violence and regular drinking. In the present study, the interaction effect of degree of exposure × gender was significant only for binge drinking, suggesting gender-specificity of this variable, possibly because of heavier drinking in response to violence exposure among boys. The interaction effects for all other substance use variables were non-significant, suggesting that although the rates of victimization and substance use varied by gender, the general patterns of increase in other types of substance use along with the levels of exposure were not gender-specific.

The role of culture in the development of adverse effects as a result of community violence exposure also remains unclear. Some argue that not only the prevalence of community violence exposure differ between cultures [5, 39], but that adverse effects of traumatic exposure may differ by culture/ethnicity and that culture plays a significant role in the expression of emotional distress [16, 17]. Others argue that trauma response knows no cultural boundaries [18]. Although the present study found significant differences between the three countries both with regard to the prevalence of violence exposure, and to the levels of substance and alcohol use, we refrain from making any direct comparisons between the study groups, as the groups differ substantially with regard to the background factors and the findings in the present study groups may not necessarily be generalizable to the respective countries as a whole. The important finding, however, was that in line with some previous studies on community violence exposure and substance and alcohol use e.g. [10], in all three countries an increase in the degree of violence exposure was similarly related to increase in substance and alcohol use. Hence, despite substantial between-county differences in levels of substance use and violence exposure, the pattern of response was similar cross-culturally.

The use of a cross-sectional design prevents us from determining causality and prospective studies are needed. The reliance of self-report on sensitive issues like violence exposure and substance use can produce recall-bias, however prior studies have indicated that self-reports on sensitive behavior by adolescents tend to be valid and reliable [40]. In fact when it comes to reporting exposure to violence adolescents themselves are the best informants, as parents often tend to underestimate the extent of violence exposure [24]. A strength of the study was the possibility to control for the participants’ own violent behavior in the multivariate analyses. Finally, the data collected in the study are over one decade old. The prevalence of the exposure to violence and of the substance use during this time could have changed, thus potentially changing the picture. However, the same pattern of associations observed in all three countries, despite the different levels of problems reported at each site, suggests that the associations would probably hold in spite the time has passed.

It is evident that community violence exposure, violence and substance abuse are interlinked. This study underline that an increase in violence exposure is associated with an increase in alcohol and substance use and that this pattern of association is neither gender nor culture bound. Hence, globally clinicians working with youth exposed to violence should assess substance use, and prevention programs aimed to reduce alcohol and substance use should address the issues of violence exposure. Finally practitioners working with delinquent youths need to take into account the effects of alcohol and substance abuse when intervening with youth at risk of violence exposure.
